# Personalized Approach in Eradication of *Helicobacter pylori* Infection

**DOI:** 10.3390/antibiotics12010007

**Published:** 2022-12-21

**Authors:** Antonio Mestrovic, Nikola Perkovic, Ante Tonkic, Zeljko Sundov, Marko Kumric, Josko Bozic

**Affiliations:** 1 Department of Gastroenterology and Hepatology, University Hospital of Split, 21000 Split, Croatia; 2 Department of Internal Medicine, University of Split School of Medicine, 21000 Split, Croatia; 3 Department of Pathophysiology, University of Split School of Medicine, 21000 Split, Croatia

**Keywords:** tailored therapy, antibiotic stewardship, *Helicobacter pylori* eradication, personalized approach, Maastricht VI guidelines

## Abstract

The increase in antibiotic resistance to *Helicobacter pylori* (*H. pylori*) is associated with a decrease in the effectiveness of eradication therapy. Although some success has been achieved by adjusting therapeutic regimens according to local data on resistance to certain antibiotics, a new approach is needed to ensure a better therapeutic response. Tailored therapy, based on sensitivity tests to antibiotics, is increasingly proving to be a superior therapeutic option, even as a first-line therapy. Moreover, the recently published Maastricht VI guidelines emphasize utilizing a susceptibility-guided strategy in respect to antibiotic stewardship as the first choice for eradication therapy. In addition, polymerase chain reaction (PCR) technology is becoming a standard tool in the diagnosis of *H. pylori* infections through non-invasive testing, which further optimizes the eradication process. We provide a review regarding the current position of the individualized approach in eradication therapy and its future prospects. Based on novel understandings, the personalized approach is an effective strategy to increase the successful eradication of *H. pylori* infections.

## 1. Introduction

*Helicobacter pylori* (*H. pylori*) infection is the main causal factor in gastritis, gastric and duodenal ulcers, gastric adenocarcinoma and mucosa-associated lymphoid tissue lymphoma (MALT) [1]. Not long after its discovery, *H. pylori* was recognized as a first-order carcinogen, and therefore by far the most significant aspect of *H. pylori* infection is its association with the development of gastric cancer [2,3].

Since the discovery of the *H. pylori* microorganism, eradication regimens have been changed and adapted many times. Triple therapy, which has been known as a standard therapy for years, has not been the therapy of first choice for some time, primarily due to insufficient eradication efficiency [4]. The main cause of this decline in eradication success is increasing antibiotic resistance rates, primarily clarithromycin, worldwide [5,6].

The modern approach to the treatment of *H. pylori* infections is based on choosing an antibiotic therapy according to the data of local antibiotic resistance. In response to the failure of triple therapy, the 2017 Maastricht guidelines proposed different models of quadruple therapy, primarily concomitant therapy and bismuth-based therapy [7]. Furthermore, different authors proposed additional modifications of the usual antibiotic combination (amoxicillin, clarithromycin, and metronidazole), such as sequential, hybrid and reverse-hybrid therapies [6,8]. The main goal of these alterations was to obtain as high of an eradication rate as possible. However, even with these modifications, the efficacy of eradication in the per protocol analyses of different randomized clinical trials is rarely above 90%, which has been identified as the main goal of antimicrobial therapy [6].

**Figure 1:**
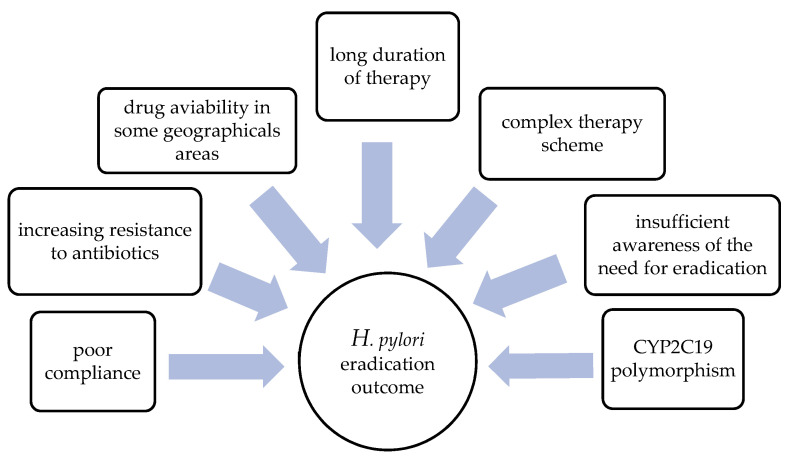
Factors affecting the efficiency of *H. pylori* eradication therapy.

The proposed therapeutic models often affect the quality of life in patients with *H. pylori* infections [8,9,10,11,12]. Namely, a large number of antibiotics and a complex regimen can have an impact on compliance and the occurrence of potentially adverse events, which can additionally limit the effectiveness of the therapy [10]. However, according to some studies, most primary eradication regimens are expected to fail in as many as 20 to 30% of cases, necessitating the need for a second-line therapy [13,14]. We can assume that this has an even more negative impact on patients’ quality of life, probably due to the complexity of therapeutic regimens and poor compliance. The main factors affecting eradication success are presented in Figure 1. 

Previous studies have shown the importance of CYP2C19 polymorphism in the effectiveness of certain proton pump inhibitors (PPI), which were, until now, an indispensable part of all eradication protocols [15]. One of the main characteristics of CYP2C19 polymorphism is the geographic diversity in its genetic differences: for example, Caucasian populations show a higher prevalence of high metabolizers compared to Asian populations [7]. 

Nevertheless, in order to achieve a suitable milieu for the action of antibiotics, it seems that there is a potential benefit in implementing the CYP2C19 genotype to guide PPI use and dosing in clinical practice [16]. 

Finally, the newly published Maastricht VI guidelines offer a different approach than the previous ones when it comes to the first line of eradication therapy [6,7]. In this review, we will present the current findings and the future prospects of the personalized approach in the treatment of *H. pylori* infections.

## 2. Current Position and Strategies

**Figure 2:**
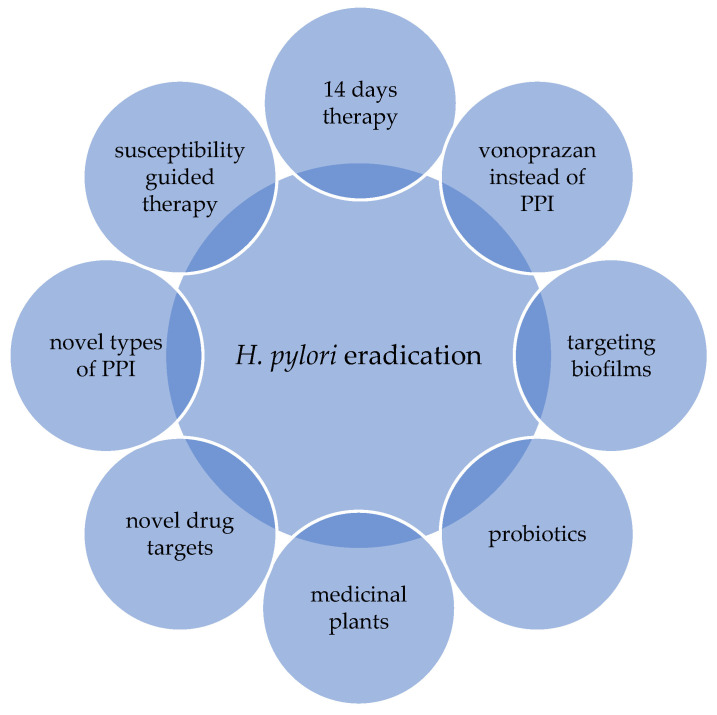
Current strategies for improving the effectiveness of eradication therapy.

There are several proposals to improve the effectiveness of the existing eradication therapy regimens. The following strategies stand out: extending the treatment length of triple therapy to 14 days; the usage of a higher dosage of PPI (proton pump inhibitors) or vonoprazan; the usage of a four-drug regimen; and supplementation with probiotics (Figure 2) [17]. 

Data from a multicenter prospective non-interventional study that included 21,533 patients from 27 European countries showed that a longer treatment duration, higher acid inhibition and compliance were associated with higher eradication rates [18]. The study revealed that European recommendations are being slowly and heterogeneously incorporated into routine clinical practice. Namely, the results of the same study indicate a reduction in the prescription of triple therapy, a longer duration of treatment and the use of a higher dose of PPI. According to the study, the above resulted in an increase in overall effectiveness (84–90%) [18]. 

Vonoprazan, a potassium-competitive acid blocker (P-CAB), has a unique pharmacological profile that provides the long-lasting inhibition of gastric acid secretion, even during night-time hours [6]. P-CAB is not dependent on the CYP2C19 genotype or the activation of parietal cells, thus providing an opportunity to improve eradication success rates even in dual therapy regimens [6]. The results of a study combining dual therapy (amoxicillin and P-CAB) showed that the eradication rate in patients with clarithromycin resistance was 95.4% [19]. At first reserved for East Asian countries, today we have studies with P-CAB therapy regimens in Western countries too, although the eradication rate has failed to reach the threshold of 90% [20]. 

A large number of studies and meta-analyses have studied the role of probiotics in the treatment of *H. pylori* infections, with conflicting results [6,21,22,23]. Current knowledge only suggests that the use of probiotics could reduce the side effects of eradication therapy, without a direct effect on *H. pylori* [6]. Such data especially refer to certain probiotics, such as *Lactobacillus* spp., *Bifidobacterium* spp. and *S. boulardii* [21,22,23]. Obviously, more studies are still necessary to assess the direct efficacy of probiotics against *H. pylori*.

Furthermore, innovative models such as plant extracts, inhibitors of biofilm formation and novel proton pump inhibitors have all been proposed (Figure 2) [17]. However, none of these proposed models have been proven as an optimal treatment option with a satisfying eradication rate. Resistance to different antibiotics remains the main obstacle to effective eradication therapy [6,17].

### Emerging Antibiotic Resistance

The resistance of *H. pylori* to antibiotics has reached alarming levels worldwide [5]. A systematic review and meta-analysis were conducted to assessed the rates of *H. pylori* resistance to commonly used antibiotics in eradication therapy in World Health Organization (WHO) regions [5]. The analysis included 178 studies, comprising 66,142 isolates from 65 countries. Increasing antibiotic resistance was observed in most WHO regions [5]. Primary and secondary resistance rates to clarithromycin, metronidazole and levofloxacin were ≥15% in all WHO regions, which is the common threshold for choosing alternative empiric regimens [5]. Exceptions were found for primary clarithromycin resistance in the Americas (10%; 95% CI, 4–16%) and the Southeast Asian region (10%; 95% CI, 5–16%), as well as primary levofloxacin resistance in the European region (11%; 95% CI, 9–13%) [5]. As expected, the resistance rates are higher in previously treated individuals than in patients who never received eradication treatment before [5]. 

Clarithromycin is still considered as a key antibiotic to eradicate *H. pylori*. However, when resistance is present, not only the probability of the treatment’s success with clarithromycin becomes very low, but it also continues to induce resistance in other bacteria [24]. Moreover, clarithromycin-resistant strains of *H. pylori* have recently been recognized by the World Health Organization as one of the 12 priority pathogens for which novel antibiotics are urgently needed [25].

In a previously mentioned study in WHO regions by Savoldi et al., resistance to clarithromycin was significantly associated with the failure of clarithromycin-containing regimens (odds ratio, 6.97; 95% CI, 5.23–9.28; *p* < 0.001) [5]. Moreover, there was a significant increase in resistance to clarithromycin in some regions, crossing the intervention threshold over 10 years: for example, the Southeast Asian region’s resistance increased from 13% in 2006–2008 to 21% in 2012–2016 [5]. Interestingly, in the same study, the pooled prevalence of clarithromycin resistance in the European region had not changed when we compared the same periods: it remained at the level of 28% [5]. 

On the other hand, Megraud et al. conducted a large prospective study regarding *H. pylori* resistance to antibiotics in Europe in 2018 and its relationship to antibiotic consumption in the community [26]. The study was performed in 24 European centers in 18 countries involving 1211 patients with positive *H. pylori* cultures. The results showed an increase in global primary clarithromycin resistance from 17.5% in 2008 to 21.4% in 2018 (*p* < 0.05) [26]. However, when we compared these results with the results of previous studies in 1998 and 2008, it is interesting to point out that the global clarithromycin resistance increased between 2008 and 2018 approximately 1% per year, thus it was not as high as we expected [26,27,28]. 

For clarithromycin resistance, there is still a large heterogeneity from country to country: for example, in Croatia there was an increase in resistance from 22% for the period of 2008–2009 to 34.6% for 2018, according to recent data [26,28]. On the other hand, in France the clarithromycin resistance rate slightly changed, from 21.3% (2008–2009) to 22.5% (2018) [26,28]. We can attribute such changes mainly to different patterns of drug prescription among countries. Some of them have taken important measures for the rational use of antibiotics, following the WHO recommendations, by decreasing the rate of antibiotic consumption, including macrolides, which are now used less for respiratory infections [26]. For example, the overall macrolide consumption decreased by 46% in France in the period from 2000 to 2015, which is obviously not the case in all European countries [26,29].

Worldwide, high metronidazole resistance has been recorded, both primary and secondary [5]. According to recent data, the highest level of metronidazole resistance is recorded in the eastern areas of the world, such as Eastern Mediterranean region (primary resistance 56%, secondary resistance 65%) [5]. However, contrary to other antibiotics, it seems that metronidazole administration in high doses and using 14-day schedules can partially overcome the resistance effect [30].

In the case of levofloxacin, increased primary resistance has also been reported, affecting the efficacy of levofloxacin-based regimens, which are designed mainly for second-line treatment, after the failure of clarithromycin-based first-line therapy [6]. However, in contrast to that of clarithromycin, the *H. pylori* resistance to levofloxacin has not increased significantly during the last 10 years (14% in 2008–2009 vs. 15.8% in 2018) [26,28]. After the failure of the standard clarithromycin, included in triple therapy, clarithromycin resistance could be expected [6]. According to the Maastricht VI guidelines, second-line eradication therapy should include levofloxacin, which has been proven to be at least as equally effective as the bismuth quadruple regimen [6,31]. It is worth highlighting the safety warning regarding the prescription of levofloxacin and its potential side effects [6]. Based on the above, it is recommended to use levofloxacin judiciously and cautiously, in situations in which the primary lines have already been exhausted.

In contrast to clarithromycin, metronidazole and levofloxacin, resistance to amoxicillin and tetracycline remained <10% in all WHO regions [5].

Increasing *H. pylori* resistance to key antibiotics, such as clarithromycin and levofloxacin, is leading to treatment failures, forcing us to use quadruple therapies, especially in Europe. Meanwhile, the overall high rate of antimicrobial consumption during the COVID-19 pandemic had a further negative impact on antibiotic resistance worldwide [32]. However, the continuation of policies of decreasing antibiotic consumption could be promising in the stabilization of resistance rates [26]. The establishment and the maintenance of local resistance monitoring systems is essential for determining guidelines and recommendations for the treatment *H. pylori* infections in specific regions, as some authors have already proposed [33].

## 3. Tailored Therapy

In order to overcome the problem of empiric therapy and improve the eradication rate, a susceptibility-guided treatment for each patient has been suggested [33,34,35]. This recommendation was finally incorporated in the recently published Maastricht VI guidelines (European *Helicobacter* and Microbiota Study Group and Consensus panel—Maastricht VI/Florence Consensus Report) [6].

According to many investigators, susceptibility-testing-guided therapy is the best strategy for increasing the eradication rate [36]. By knowing the prevalence of antibiotic resistance in a certain population, we can predict efficacy of different therapy models in susceptible and resistant strains of *H. pylori* microorganisms [37,38].

The selection of a therapy based on information about the antibiotics that have been used previously represents the origin of the personalized approach in the eradication of *H. pylori* infections [35]. Romano et al. conducted a prospective study on 401 subjects in a region with high resistance to clarithromycin comparing bismuth quadruple therapy with concomitant therapy; concomitant therapy was prescribed only to those subjects who had not previously used clarithromycin, based on their medical history data [39]. The results showed a high but similar eradication efficiency of concomitant therapy and bismuth-based therapy in patients who had not previously taken clarithromycin: 88.2% vs. 91.5% (*p* = 0.26) in the ITT analysis and 91.2% vs. 95.8% (*p* = 0.07) in the PP analysis [39]. The study was an example of how to simply avoid unnecessary proscriptions of macrolides in order to rationalize the use of antibiotics in regions with high resistance to clarithromycin.

Already, therapies based on antibiotic susceptibility testing seem to be an optimal option in reducing and optimizing eradication therapy. The results of a meta-analysis conducted by Chen et al., consisting of 13 controlled clinical trials and including 3512 participants, showed higher eradication rates when tailored therapies were used, compared to empirically chosen regimens [40]. Recently, Mao Q. et al. in a meta-analysis involving 21 studies showed that antibiotic-guided therapy yielded a significantly better efficacy rate compared to that of empirical regimens in first-line treatments (RR, 1.14 (95% CI, 1.08–1.21), I2 = 72.2%) [41]. However, in the second line, they did not find any difference in eradication outcomes (RR, 1.05 [95% CI, 0.84–1.30], I2 = 80.6%), suggesting that empirical therapy could be as efficient as tailored therapy. Still, the results of the study should be interpreted with caution due to the high heterogeneity of the evidence in the research [41].

Gingold-Belfer et al. performed a systematic review and meta-analysis of 16 randomized controlled trials, including 2374 patients who received susceptibility-guided therapy and 2451 patients who received empirical treatments [42]. The results showed that susceptibility-guided treatment may be slightly superior to empirical first-line triple therapy in naive patients. However, susceptibility-guided treatment did not appear to be superior to empirical first-line quadruple therapy or empirical rescue therapy. The superiority of susceptibility-guided therapy to empirical triple therapy with clarithromycin was found only in cases in which the clarithromycin resistance exceeded 20% (RR, 1.18; 95% CI, 1.07–1.30; *p* = 0.001, I2 = 81%) [42].

Finally, tailored therapy might allow for the prescription of optimized clarithromycin-based triple therapy to patients with clarithromycin-susceptible strains in areas with high overall clarithromycin resistance. It should be emphasized that triple therapy with clarithromycin can be used in areas with high clarithromycin resistance rates only with a previous antibiotic sensitivity test [6]. The global rate of primary clarithromycin resistance is >20%, based on the results of the previously mentioned prospective study conducted in 18 European countries involving 1211 *H. pylori* culture-positive patients [26]. Accordingly, more than two-thirds of our patients, respecting regional differences, are eligible for clarithromycin-based first-line eradication therapy. By selecting proper patients, we can proscribe standard triple therapy in cases of clarithromycin-sensitive strains, and thus avoid unnecessary quadruple regimens.

Table A1 shows the randomized clinical trials published in the last 10 years, comparing tailored and empirical therapies in first-line eradication treatment [12,43,44,45,46,47,48,49,50,51]. Although tailored therapy is widely considered as superior to empirical therapy in first-line treatment, the overall results are diverse, in which some studies showed no difference at all between the two therapy groups. However, the heterogeneity of the study’s protocols and reporting should be accounted for [52].

## 4. Anything New in Diagnostics?

Traditionally, diagnostic tests for *H. pylori* infections have been divided into noninvasive and invasive ones [6]. However, PCR technology has found its place in both groups, as it can be performed from a gastric biopsy sample and stool, respectively [6,7]. Among noninvasive tests, the ^13^C-urea breath test (UBT) and stool antigen test (SAT) have been the main tools in clinical practice, both for the diagnosis and confirmation of eradication [6]. IgG serology is not able to differentiate acute infection from past infection, and therefore it is not suitable as a confirmation test [6]. Among invasive tests, the rapid urease test (RUT), histology staining and culture confirmation stand out.

The ^13^C-UBT is still considered to be a standard tool in practice and studies. More recent data have shown some benefits to using citric acid over other meals in order to improve accuracy and sensitivity [53]. Citric acid helps slow gastric emptying and thus increases the contact time with *H. pylori* urease [53,54].

The SAT became a suitable diagnostic tool in practice before and after eradication [6]. It is important to highlight that tests based on monoclonal antibodies are superior in comparative studies to polyclonal tests [55]. SATs using enzyme immunoassay (EIA) test kits should be preferred as they have shown better performance than rapid immunochromatography tests [55,56].

When endoscopy is indicated, the RUT represents a fast, cheap and reliable tool for *H. pylori* diagnosis [6,57]. Although a highly specific test (95–100%), its sensitivity (85–95%) may be affected by small sample sizes, as less than 104 bacterial cells can lead to false-negative results [57,58,59]. The Maastricht VI guidelines suggest the practical reuse of RUT test slides which are meant to be discarded after reading them [6]. Instead of taking additional biopsies for PCR or other tests, those already taken for the RUT can be reused for the purpose of PCR analysis [60,61]. Analyses have shown a high correlation between the reuse of these kinds of test samples [60].

In dyspeptic patients older than 50 years, an upper-GI endoscopy is required, regardless of whether they have alarming symptoms [6,7]. When an endoscopy is indicated, it should involve biopsy sampling, as recommended by the guidelines [6]. A biopsy analysis should result in an etiological diagnosis, gastritis staging and *H. pylori* status [6]. Gastritis histological staging should be assessed through the OLGA (Operative Link on Gastric Atrophy) and OLGIM (Operative Link on Gastric Intestinal Metaplasia) systems [62,63].

The main limitation of the presented non-invasive diagnostic methods is that they can solely detect *H. pylori* but cannot provide information on the drug susceptibility of the bacterium. For that purpose, we use antibiotic sensitivity testing.

### Antibiotic Sensitivity Testing Issues

Antibiotic sensitivity testing is mainly performed by the following two methods: *H. pylori* cultures and genotyping by molecular detection [41]. In the first, *H. pylori* is cultured from a gastric mucosa sample following a biopsy, while the antibiotic sensitivity is detected by agar dilution, disk diffusion or an E-test [41]. Among them, the agar dilution method is considered the gold standard, although it is costly, time-consuming and labor intensive (2–4 weeks) [35,64]. Furthermore, the success rate of cultures and susceptibility testing ranges from 75 to 90% [36,65]. The second method is genotyping by molecular detection (real-time PCR and fluorescence in situ hybridization) from stool and stomach biopsy specimens, whereas point mutations associated with resistance to specific antibiotics are usually detected through commercially available kits [42]. In addition, high-throughput whole-genome sequencing techniques have been used to identify drug-resistant mutations [66,67]. Today, molecular methods (in particular, real time-PCR, whole-genome sequencing and digital PCR) are being improved more and more, thus allowing for the detection of *H. pylori* mutations associated with resistance to the commonly used antibiotics in eradication regimens with clarithromycin, levofloxacin, tetracycline and rifampicin [6].

The mechanisms of *H. pylori* bacteria resistance against the antibiotics that are mainly used for eradication are now largely familiar [68]. For example, resistance against clarithromycin is largely because of mutations in the 23S rRNA gene [6,38]. Furthermore, only a few mutations (A2143G, A2142G and A2142C) are responsible for almost all cases of clinical resistance [68]. On the other side, for levofloxacin, data have shown that resistance is mostly due to point mutations in the gyrase gene gyrA [67,69,70]. For tetracycline and rifampicin, fewer data are available; however, according to a known fact, resistance to tetracycline is mostly due to mutations in 16SvrRNA genes and to rifampicin due to mutations in the RNA polymerase gene rpoB [68]. Resistance to amoxicillin, as a basic antibiotic in most of the eradication regimens, is rare, but complex [6]. Moreover, raising resistance to metronidazole is highly complex [6]. Furthermore, metronidazole resistance in vitro may not correspond to what happens in vivo and bismuth-containing therapy may overcome metronidazole resistance [6,24]. Thus, a lot is expected from whole-genome or focused next-generation sequencing, which could predict antibiotic resistance phenotypes with more precision, even in more complicated cases, such as metronidazole or amoxicillin resistance [6].

Despite technological advancements in the PCR diagnostics of *H. pylori* infections, the successful integration and application of a susceptibility-guided strategy will depend on the rapidity of the spread and acceptability of these methods in clinical practice.

## 5. Novelties of the Maastricht VI Guidelines

The latest Maastricht VI guidelines from 2022 recommended routinely preforming susceptibility tests (molecular or culture) if available, even before prescribing first-line treatment, in respect to antibiotic stewardship [6]. However, how this strategy will be used in practice remains to be established. For now, it remains a matter of debate whether it is necessary to subject every patient to an upper endoscopy to provide the sample needed to determine antibiotic susceptibility. We need to point out that determination of antibiotic susceptibility is not only benefit for the specific patient, but it also provides the possibility of evaluating the prevalence of antibiotic resistance in naive patients. In this way, we can create and update data on local resistance to antibiotics and contribute to the formation of optimal and effective eradication regimens in a certain region.

Although in theory the recommended approach, based on susceptibility-guided therapy, seems ideal, it is often difficult to implement it in clinical practice. Upper endoscopy is expensive, invasive, often uncomfortable for the patient, and in the end, not even necessary in all patients with dyspepsia [6].

So far, the non-invasive ‘test and treat’ strategy for patients with dyspepsia has proven to be an acceptable method of selection [71]. In practice, non-invasive methods for the confirmation of *H. pylori* infections are mostly used, such as the stool antigen test and urea breath test [7]. Nevertheless, a positive result, in accordance with the recent Maastricht recommendations, necessitates that the antibiotic resistance is determined [6]. In theory, this would require an endoscopic examination with a sample collection, which ultimately multiplies tests with a questionable cost–benefit effect.

However, recently, non-invasive methods have been developed, such as PCR test from the stool, which makes it possible to detect antibiotic susceptibility and to avoid the invasiveness of an upper endoscopy [72]. A recent meta-analysis showed further progress in DNA extraction methods from stool samples [73]. The study’s results proved there is a high diagnostic accuracy for detecting clarithromycin resistance in *H. pylori*-positive patients with a sensitivity of 91% and specificity of 97% [73].

The COVID pandemic certainly contributed to the wider use of PCR technology in everyday practice. Thus, real-time PCRs are available and used in most laboratories worldwide [6].

Recently, Japanese authors published the results of a study in which Smart Gene™, a method based on the concept of point-of-care genetic testing, was used [74]. The results showed that the detection performance of Smart Gene™ was comparable with that of real-time PCR and sequencing analyses [74]. Although it was not compared with cultures, mentioned method is considerably shorter than real-time PCR tests, thus making it more appropriate for everyday practice [74].

## 6. CYP2C19 Polymorphism

CYP2C19 is the main enzyme in the metabolism of PPI [75]. Furthermore, the effects of PPI are determined by the activity of metabolic enzymes, cytochrome P450 enzymes and CYP2C19 with genetic differences (the homozygous EM (HomEM), heterozygous EM (HetEM) and poor metabolizer)) [76]. Thus, the genetic background of a patient and the pharmacological profile of drug can impact the decision whether we should use different PPIs and/or increase doses of PPIs. CPY2C19 genotyping can provide a useful way to further optimize eradication therapy, in that the CYP2C19 genotype is related to the different abilities of PPIs to inhibit gastric acid secretion [6,15].

A difference between Caucasian and Asian subjects, regarding the frequency of genetic deficiencies, has been recorded [77]. Nearly 20% of the Japanese population lacks CYP2C19 activity, but on the other hand only 3% to 4% of Caucasian population are poor metabolizers [77]. Furthermore, the prevalence rate of HomEM is about 70% for Caucasians, but only 30–40% for Asians [78]. Therefore, geographic differences should be considered when selecting a proton pump inhibitor or its dose for eradication treatments.

A homozygous extensive metabolizer (HomEM) is distinguished by two wild-type alleles (or *1/*1) [79,80]. However, a heterozygous EM (HetEM) is characterized by carrying one loss-of-function (LOF) variant allele (frequently *2 or *3), as opposed to a poor metabolizer (PM), in which two LOF variant alleles are found (*2/*2, or *2/*3) [79,80]. A HomEM metabolizes the PPI at high rates by producing excessive amounts of the enzyme [75]. A HetEM, with one wild type and one mutation type, metabolizes the PPI at moderate rates [75]. The differences between the metabolizers have been demonstrated by studies [81]. The results of a meta-analysis performed by Padol et al. showed a significant difference in *H. pylori* eradication rates between wild-type individuals and carriers of at least one LOF allele (OR = 2.26; 95% CI: 1.58–2.96; *p* < 0.0001) [81]. A significant difference was also demonstrated between HomEM and HetEM individuals (OR = 2.79; 95% CI: 1.77–4.41; *p* < 0.0001) [81]. However, another study analyzed individual agents separately, with significant differences only for omeprazole and lansoprazole, whereas no differences between the genotypes were observed for rabeprazole [82].

However, it is reasonable to assume that increasing the dosage might overcome the problem of CYP2C19 polymorphism. A randomized, open-label study demonstrated that increasing the dosage of omeprazole (from 20 to 40 mg daily) would improve the efficacy of eradication, both in homozygous and heterozygous extensive metabolizers [83]. However, other studies have shown contrary results regarding dose-dependent effectiveness using omeprazole, rabeprazole and lansoprazole [84,85]. Therefore, this strategy needs further analysis.

Another possible method might be performing CYP2C19 genotyping before starting eradication therapy. However, such routine examinations are expensive and are not always available. A meta-analysis including six studies with a total of 1703 patients showed that high-dose PPIs (twice the standard dose) increase cure rates by 6–10% in comparison with standard doses in seven-day triple therapy [86]. Possible strategies to avoid CYP2C19 genotype issues could be: (1) selecting PPIs metabolized by the non-enzymatic pathway or; (2) considering increasing the dose of CYP2C19-sensitive PPIs [75].

## 7. Future Prospects in the Approach to *H. pylori* Infections and Treatment

Until now, all valid guidelines have defined an *H. pylori* infection as an infectious disease that should be treated regardless of the presence of symptoms [6,87,88]. Considering the known, long-term consequences of *H. pylori* infections, it is to be expected that attitudes about the need to treat these infections will remain unchanged in the future.

The increase in antibiotic resistance limits the number of available empiric therapies, which highlights the need for changing the current approach. Despite numerous attempts and proposals to change therapies, none have so far produced the expected increase in the eradication rate. Nevertheless, targeted therapy in the first line of eradication, based on a previous sensitivity test, represents a reliable and directed therapeutic approach. Eradication rates of targeted therapies often exceed 90%, which is considered successful.

With the exception of cultures and the classic determination of sensitivity to certain antibiotics, PCR technology has brought about new dynamics in diagnostics, which enables the detection of bacteria and resistance to certain antibiotics. In fact, today a PCR is possible not only from a stomach mucosa sample, but also from a stool sample; this makes the process of diagnosing and obtaining targeted information about resistance simpler and more accessible.

However, the use of tailored therapy is largely limited by the availability of the necessary tests, as well as the capabilities of local health systems. To that extent, awareness of the rational use of antibiotics is necessary in everyday work, for example avoiding prescribing clarithromycin to anyone who has taken it before. The extension of the empiric regimen to 14 days, the use of PPIs in a higher dose and the promising role of vonoprazan offer a more optimistic picture for future treatment approaches. Additionally, the discovery of new, broader-spectrum antibiotics, as announced by the WHO, remains imperative.

On the other hand, a completely new approach to *H. pylori* infections has been made through nanotechnology [89]. Drugs used in eradication treatments have many obstacles on their pathway to the site of action, which can seriously undermine their effectiveness [90]. A possible way to overcome these problems might be emerging nanomedicine based on a nanotechnological approach that may enable the efficient and effective targeted delivery of drugs [90]. The use of nanoparticular systems can be beneficial in the prevention of drug degradation in the gastric lumen, as well as in the optimal medication delivery to highly colonized *H. pylori* areas in the stomach [91]. Compared to antibiotics, nanoparticular systems are proving not only to be less toxic, but also to have a role in decreasing the bacterial resistance against antibiotics [92].

With the recent advancements in the molecular description of *H. pylori* pathogenesis, more studies are needed to examine the practical application of nanotechnology in eradication treatments [93]. In Table 1, the possible directions of the personalized approach in the treatment of *H. pylori* infections are shown.

## 8. Conclusions

The personalized approach in *H. pylori* eradication treatments reduces unnecessary antibiotic prescription and has a positive effect by limiting the emergence of antibiotic resistance worldwide. Additionally, it allows us to form resistance surveys over time. However, it should be noted that the use of tailored therapy may be limited by availability and also sustainability in some areas. In our opinion, future treatment recommendations for the eradication of *H. pylori* infections should be based on a personalized approach whenever possible.

## Figures and Tables

**Table 1:** Future directions of personalized approach in treatment of *H. pylori* infection.

susceptibility-guided therapy in first-line treatment
rationale empiric first-line treatment (e.g., avoiding clarithromycin if used previously)
high-dose IPP
novel antibiotics
14-day therapy
vonoprazan instead of PPI
PCR in diagnostics (from stool sample, as non-invasive test)
use of nanotechnology

## Data Availability

Not applicable.

## Appendix A

**Table A1:** Randomized clinical trials comparing tailored and empirical therapy.

Authors	Year	Country	N	Susceptibility Testing	Tailored Therapy		Empirical Therapy		Results Tailored vs. Empirical	Comments
					Regime (PPI Included)	Duration (Days)	Regime (PPI Included)	Duration (Days)		
Delchier et al. [43]	2019	France	526	GenoT ype HelicoDR^®^ PCR	AM (1000 mg bd) + CL (500 mg bd) AM (1000 mg bd) + LE (250 mg bd) AM (1000 mg bd) + MT (500 mg bd)	7–14	AM (1000 mg bd) + CL (500 mg bd)	7	ITT: 85.5% vs. 73.1%, *p* = 0.003 PP: 74.4% vs. 86.5%, *P* = 0.003	tailored therapy superior
Dong et al. [44]	2015	China	90	Culture with E test and PCR	AM + CL + BI AM + BI + LE AM + BI + FU BI + CL + MT (doses n/a)	7	BI (600 mg bd) + AM (1000 mg bd) + CL (500 mg bd)	7	ITT: 91.1 % vs. 73.3%, *p* = 0.027 PP: 96.3% vs. 78.6%, *p* = 0.021	tailored therapy superior
Zhou et al. [40]	2016	China	1050	Culture with E test	AM (1000 mg bd) + CL (500 mg bd) AM (1000 mg tid) + TI (500 mg tid)	10	AM (1000 mg bd) + CL (500 mg bd) + BI (220 mg bd) AM (1000 mg bd) + CL (500 mg bd) + TI (500 mg bd)	10	ITT: 88.7% vs. 77.4% vs. 78.3 %, *p* < 0.001 PP: 93.3% vs. 87.0% vs. 87.4%, *p* = 0.021	tailored therapy superior than triple plus bismuth and concomitant groups
Chen et al. [46]	2019	China	382	Culture with agar dilution	AM (1000 mg bd) + CL (500 mg bd) AM (1000 mg bd) + MT (400 mg bd) AM (1000 mg bd) + LE (500 mg bd) AM (1000 mg bd) + BI (600 mg bd) + MT (400 mg qid)	14	AM (1000 mg tid) + MT (400 mg tid) + BI (600 mg bd)	14	ITT: 91.6% vs. 85.4%, *p* = 0.12 PP: 97.7% vs. 97.6%, *p* = 1.00	no significant difference
Ong et al. [47]	2019	South Korea	397	Culture with agar dilution and PCR	AM (1000 mg bd) + CL (500 mg bd) AM (1000 mg bd) + MT (500 mg bd)	14	AM (1000 mg bd) + CL (500 mg bd) + MT (500 mg bd)	14	ITT: 86.2% vs. 81.6%, *p* = 0.132 PP: 90.2% vs. 86.5%, *p* = 0.179	no significant difference
Pan et al. [48]	2020	China	467	Culture with agar dilution	AM (1000 mg bd) + CL (500 mg bd) AM (1000 mg bd) + LE (200 mg bd) AM (1000 mg bd) + FU (100 mg bd) BI (200–220 mg bd) + AM (100 mg bd)/CL (500 mg bd)/LE (200 mg bd)/FU (100 mg bd) *	14	AM (1000 mg bd) + CL (500 mg bd) + BI (200–220 mg bd)	14	ITT: 85.99% vs. 67.32% vs. 63.69%, *p* < 0.001 PP: 91.22% vs. 74.64% vs. 68.49%, *p* < 0.001	no significant differences between tailored triple therapy and empirical bismuth therapy tailored bismuth therapy was the most efficacious regimen
Perkovic et al. [12]	2021	Croatia	80	Culture with E test	AM/CL/MT/ TT/LE * (doses n/a)	14	AM (1000 mg bd) + CL (500 mg bd) + MT (500 mg bd)	14	ITT= 92.5% vs. 70%, *p* = 0.010 PP = 100% vs. 87.5%, *p* = 0.030	tailored therapy superior
Cho et al. [49]	2021	South Korea	282	dual priming oligonucleotide-based PCR	PPI + AM + CL PPI + TT + MT + BI (doses n/a)	7	AM (1000 mg bd) + MT (750 mg bd) + BI (600 mg bd)	14	ITT: 80.9% vs. 85.8%, *p* = 0.262 PP: 89.0% vs. 93.5%, *p* = 0.198	empirical bismuth therapy exhibits similar efficacy and improved cost-effectiveness compared to tailored
Choi et al. [51]	2021	South Korea	217	dual-priming oligonucleotide-based PCR	MT (500 mg bd) + BI (300 mg qid) + TT (500 mg qid) AM (1000 mg bd) + CL (500 mg bd)	10 - 14	AM (1000 mg bd) + CL (500 mg bd) + MT (500 mg bd)	10	ITT: 82.7% vs. 82.2%, *p* = 095 PP: 90.1% vs. 91.6%, *p* = 0.72	no significant difference in eradication rates; adverse events were significantly lower in tailored group
Kim et al. [50]	2022	South Korea	290	dual-priming oligonucleotide-based PCR	AM (1000 mg bd) + CL (500 mg bd) MT (500 mg tid) + BI (120 mg qid) + TT (500 mg qid)	14	AM (1000 mg bd) + CL (500 mg bd) + MT (500 mg bd)	14	ITT: 85.5% vs. 82.8%, *p* = 0.520 PP: 94.6% vs. 88.6%, *p* = 0.084	empirical and tailored therapy showed similar overall eradication rates

* Two of the listed antibiotics were given, based on the susceptibility testing results. Abbreviations: PPI, proton pump inhibitor; AM, amoxicillin; CL, clarithromycin; MT, metronidazole; LE, levofloxacin; BI, bismuth; FU, furazolidone; TI, tinidazole; TT, tetracycline; PCR, polymerase chain reaction; ITT, intention-to-treat; PP, per-protocol; bd, twice a day; tid, three times a day; qid; four times a day; n/a, not available.
